# See Elegans: Simple-to-use, accurate, and automatic 3D detection of neural activity from densely packed neurons

**DOI:** 10.1371/journal.pone.0300628

**Published:** 2024-03-22

**Authors:** Enrico Lanza, Valeria Lucente, Martina Nicoletti, Silvia Schwartz, Ilaria F. Cavallo, Davide Caprini, Christopher W. Connor, Mashel Fatema A. Saifuddin, Julia M. Miller, Noelle D. L’Etoile, Viola Folli

**Affiliations:** 1 Center for Life Nano- and Neuro-Science@Sapienza, Istituto Italiano di Tecnologia (IIT), Rome, Italy; 2 D-tails s.r.l., Rome, Italy; 3 Department of Engineering, Campus Bio-Medico University, Rome, Italy; 4 Department of Anesthesiology, Perioperative and Pain Medicine, Brigham and Women’s Hospital, Harvard Medical School, Boston, MA, United States of America; 5 Department of Cell and Tissue Biology, University of California, San Francisco, San Francisco, CA, United States of America; Cardiff Metropolitan University, UNITED KINGDOM

## Abstract

In the emerging field of whole-brain imaging at single-cell resolution, which represents one of the new frontiers to investigate the link between brain activity and behavior, the nematode *Caenorhabditis elegans* offers one of the most characterized models for systems neuroscience. Whole-brain recordings consist of 3D time series of volumes that need to be processed to obtain neuronal traces. Current solutions for this task are either computationally demanding or limited to specific acquisition setups. Here, we propose See Elegans, a direct programming algorithm that combines different techniques for automatic neuron segmentation and tracking without the need for the RFP channel, and we compare it with other available algorithms. While outperforming them in most cases, our solution offers a novel method to guide the identification of a subset of head neurons based on position and activity. The built-in interface allows the user to follow and manually curate each of the processing steps. See Elegans is thus a simple-to-use interface aimed at speeding up the post-processing of volumetric calcium imaging recordings while maintaining a high level of accuracy and low computational demands. (Contact: enrico.lanza@iit.it).

## Introduction

Neuroscience seeks to unravel the relationship between neural dynamics and behavior, greatly aided by advanced imaging techniques that allow for single-cell resolution of brain activity [1–4]. The nematode *Caenorhabditis elegans*, with its fully mapped yet not entirely understood nervous system, is an ideal model for these studies [5–7]. Although producing mainly graded potentials, the transparency and genetic tractability of *C. elegans* contribute to its effectiveness as a model organism [8–10], particularly useful for studying human diseases and their effects on neuronal dynamics [11].

Neuron tracking and segmentation challenges are addressed using methods ranging from traditional 3D blob segmentation to deep learning, each with its own limitations [12–15]. Interframe neuron tracking can either be sequence-dependent, prone to accumulating errors, or independent, with its effectiveness hinging on the accuracy of the base model assumptions.

[13, 16–22]. For neuron annotation, automated methods exploit stereotyped neuronal position [3, 5, 6, 23–25], but these may be subject to artifacts and variability. Using fluorescent identity markers is an alternative, albeit it demands more requirements on sample preparation and optical setups [26, 27].

We introduce See Elegans, an algorithm for automatic detection, tracking, and identification of *C. elegans* neurons, incorporating both sequence-dependent (as in [12, 14, 28]) and sequence-independent methods (as in [13, 29]) to track cells undergoing limited arrangement deformations. It provides advantages over existing software like Trackmate [30] and ROIedit3D [14], as it uses the position of detected cells to locate neurons below the detection threshold and eliminates the need for the RFP channel for tracking. See Elegans performs automatic identification of about 20 neurons based on soma position, neuronal activity, and coherence. The GFP channel is sufficient for neuron identification, eliminating the need for further genetic modifications and complex optical setups. See Elegans demonstrated better performance compared to other publicly available algorithms, while it demonstrated high accuracy in neuronal identification of backward locomotion promoting neurons, with promising results on forward locomotion promoting ones. While the algorithm automates the core processes of detection, tracking, and neuron identification, it also includes a human-in-the-loop approach for initial parameter settings, thereby enhancing the adaptability of See Elegans to varied data sets and experimental conditions. Its user-friendly interface allows for efficient supervision, parameter adaptation, and result curation, significantly simplifying the process of extracting neuronal traces from calcium imaging recordings of *C. elegans*. The integration of these features makes See Elegans an easy-to-use and accurate tool, optimizing both the precision and efficiency of calcium imaging data processing.

## Design and implementation

To obtain activity traces and neuronal identities from raw images, See Elegans involves three steps: (1) cell segmentation, (2) tracking, and (3) identification. In the cell segmentation step, the algorithm detects cells in the image based on their size, shape, and intensity. Next, in the tracking step, the algorithm tracks the cells across time frames to obtain activity traces for each cell. Finally, in the identification step, the algorithm assigns identities to the cells based on their activity patterns. These three steps may be run separately, and their outputs can be used as inputs for downstream analysis. Further details can be found in the Materials and Methods section. The following sections report a description of the main characteristics of each of these steps.

### Cell segmentation

The detection process allows the user to locate neuronal spots by convolving each volume of the acquisition with a 3D laplacian of gaussian filter (log filter), as in TrackMate, based on [31], and then by thresholding the image resulting from the element-wise product between the filtered image and the original one. The threshold, the size of the log filter, and the variance of its gaussian are user-defined. As visual feedback, a simple interface shows a real-time preview of the ROI centers found for the chosen parameter set.

### Tracking

The tracking process is divided into two steps. The first step allows the user to apply the Runge-Kutta algorithm to solve the linking problem representing it as a linear assignment problem (LAP), as in [30, 31]. A simple interface allows the user to select a time crop of the acquisition to test the parameters for the LAP tracking: maximum distance, maximum time gap between spots, and non-linking costs. Once the parameters are set, the user can run the tracking step on the whole video. Alternatively to TrackMate, See Elegans also retrieves segment links based on the relative distance between them across all frames (see Methods), independently from their order. This first part of the tracking process is suited to compensate for rigid body movements, with a sequence-dependent method (Runge-Kutta algorithm) and a sequence-independent one (based on relative distances). However, when neurons fall under the detection threshold, they cannot be linked. To compensate for such situations, the second step calculates the position of the missing neuron, taking into account its position at the previous frame with respect to 20 of its closest neighbors, similarly to [28]. So, if neuron A is missing in frame *i* but is visible in frame *i* − 1, and *N* is the set of its closest neighbors, then its position in frame *i*, $p_{A_{i}}$, is calculated according to the following equation:

$$\begin{array}{c} p_{A_{i}}=\frac{1}{N}\sum_{J\in N}p_{J_{i}}-p_{J_{i-1}}+p_{A_{i-1}} \end{array}$$ (1)

**Fig 1:**
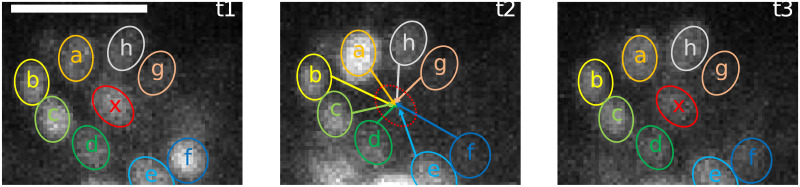
ROI falling under detection threshold and position reconstruction. The images show the temporal progression (t1-t3) of an ROI from data set 2. Neuron x is visible at the center in t1 and t3, but not detected at t2. Its position is inferred from its nearest neighbors (a-h) in previous frames. The white bar represents 10 *μ*m.

In this way, the position of the neuron in the frame where it is missing is inferred through the sum of the average position of 20 of its closest neighbors and the average distance between them and the missing neuron detected in the previous frame. Fig 1 shows an example of the position reconstruction based on a few neighboring spots and on Eq 1. See Elegans uses this position inference technique also for the manual addition of neurons that cannot be automatically detected. This step occurs after the tracking process because it relies on full traces to reconstruct the trace of a missed spot. The user is asked to inspect the result of the tracking process and to annotate by hand the position of undetected neurons at any time during the recording where it is relatively well visible. The algorithm treats the added spot as a track that has only one time point and reconstructs it following the same steps described above. It is worth noticing that in its current form, the proposed algorithm can account for small deformations in the arrangement of the neurons but fails to properly track spots in the presence of significant mutual-position changes, as when the nematode strongly bends its nose. In particular, when a neuron undergoes movement exceeding the displacement threshold set in the second step and simultaneously falls below the detection threshold, the algorithm is faced with a potential ambiguity upon the neuron’s re-emergence. In such instances, there is a possibility that the neuron might be erroneously registered as a novel entity, or it could inadvertently impact the tracking of another neuron within its vicinity. Conversely, if a neuron’s reappearance occurs within the threshold, or if it traverses beyond the threshold but remains within detectable limits, the algorithm is designed to merge the respective tracking segments. This approach is particularly advantageous for reconstructing neuron positions, as it utilizes spatial information relative to adjacent neurons, thereby enhancing the accuracy of the neural trace, especially in scenarios where signal detection is intermittent.

### Identification

Of the 302 neurons in *C. elegans*, about a third are located in the head. See Elegans allows identifying a subset of key head neurons based on their position and signal correlation. As described in [28], neuronal calcium signals of constrained *C. elegans* recorded over ten-minute-long acquisitions show a stereotyped activity that can be reduced in dimensionality through PCA. In the axes decomposition, there are three groups of neurons that dominate over the rest in the absence of stimuli: neurons whose activation is associated with backward movement, neurons associated with forward movement, and neurons that activate during turns. Although recent works showed how neural correlations may change from constrained to unconstrained animals, some of the neuronal correlation properties still persist in moving ones [32] and can thus be useful to help with neuronal identification. Such stereotyped activity has been reported in multiple works ([28, 33–37], some of which identification has been performed also on the basis of location and fluorescent signals). The identification step only requires the user to specify the anterior direction (i.e. the position of the nose tip in the recording), which is used by the algorithm to initially identify 6 candidates for the AVA, AVE, and AIB pairs. These neurons are expected to be close, symmetrically arranged [5], and with a high correlation among them [28]. Moreover, they are usually clearly visible and easy to recognize by their activity and position. The algorithm thus looks for a set of neurons that best fits these requirements and once it finds it, it creates a new coordinate system for the visualization and for further processing. In this system, the x, y, and z axes follow the anterior, dorsal and left directions respectively. All coordinates are divided by the average distance among the six identified neurons to compensate for scale differences, and the origin of the coordinate system is set to the mean point of the six neurons initially identified. The neuronal arrangement thus obtained is compared with a model distribution based on [5] in a coordinate system with the same scaling. Neuronal spots of the model are assigned to the observed ones by treating the task as a linear assignment problem again. However, this time the cost function between two neurons takes into account not only their position, but also their correlation and some additional rules derived from previous experiments and reported experiments: following the work of [28], the six correlated neurons may generally be identified as AVA, AVE, and AIB pairs [4, 38–41]. Other identifiable neurons correlated with these ones are RIM pairs [40] and the VA and DA motor neurons [42–44]. The activity of all these neurons has been reported to be correlated with backward locomotion [38, 39, 42]. Another identifiable set of correlated neurons that are in anti-correlation with the first group includes the following neurons: RME [4], RID [45], RIB [46], AVB [4], VA [38], and DB [38, 43, 44] neuronal classes, whose activity is associated with forward locomotion. With these identification rules, the algorithm makes a guess on the identity of the neurons, which can be then modified by the user. It is worth noticing that, in conditions under which normal neuronal correlation is hindered, the user has to manually assign the identities of the affected neurons. Additionally, this process is strongly dependent on the identification of two pairs of three neurons co-activating more than once during the video (namely AVA, AVE, and AIB). Failing this initial step may result in a high rate of erroneous identifications. However, the interface allows the 3D visualization of subgroups of neurons showing high/low anti-/correlations with respect to a neuron of choice, thus providing visual help to the user during manual annotation. Once a few neurons are identified, these may be used as reference points for the comparison with the model distribution of neurons for a new guess about the class identity of the spots.

## Results

To measure the performance of the previously described steps, we applied See Elegans to three data sets and compared its output with other available tracking software. See Elegans can handle a variety of image types, including 2D and 3D stacks. The time intervals between frames can vary, and the system can process images with animals that are relatively stationary, with minimal movement. This is the typical case of many publications [28, 47–49]. To highlight the robustness and versatility of the proposed method, the data sets have been recorded with different instruments and techniques (see Methods) in three laboratories: the first two sets of data are acquired through spinning-disk confocal microscopy using different acquisition devices, while data set 3 was obtained through light-sheet microscopy applied to a nematode previously treated with tetramisole but without microfluidic chip confinement for movement blocking, and characterized by a lower signal-to-noise ratio on the focal plane compared to the first ones, representing a challenging scenario. Regarding animal movement, it is important to notice that it may impair the ability of the algorithm to track neurons across time. In particular, in the case of non-paralyzed nematodes, it is crucial to specify the maximum distance that a neuron can travel from one frame to another in the tracking step, to mitigate cross-frame linking errors.

### Segmentation

**Fig 2:**
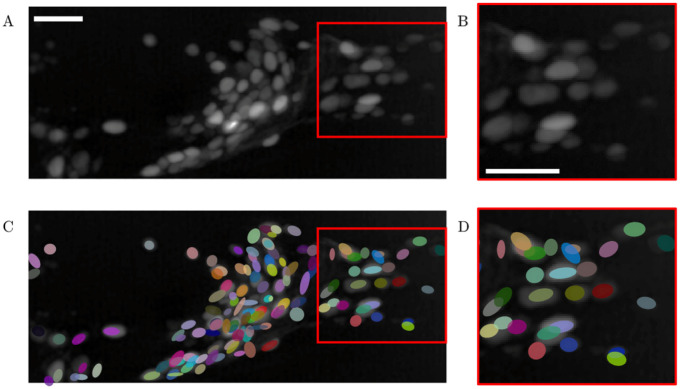
Maximum projection of a volume recorded from a *C. elegans* head and result of the segmentation. Panel A is a maximum projection of a volume centered on a nematode head acquired in the GCaMP channel (data set 1). Panel C reports the result of the detection process of the proposed algorithm. Panels B and D report a zoom-in of the first two panels respectively. White bars represent 10 *μ*m.

To show the performance in neuron detection, Fig 2 reports a maximum projection of data set 1, and the result of cell segmentation using See Elegans together with a crop outlined in red. Additionally, for each data set, we determine the performance of See Elegans by counting the number of true positives, false negatives, and false positives resulting from the automatic detection, and then calculating their rate and accuracy according to the formula:

$$\begin{array}{c} Accuracy=\frac{TP}{TP+FP+FN}, \end{array}$$ (2)

where TP, FP, and FN are true positives, false positives, and false negatives respectively as in [14]. We first determine the ground truth through manual annotation of all neuronal soma centers by examining each slice of one volume from each data set, then we count as a true positive any blob detected within 3 *μ*m from a manually annotated neuronal soma. If a spot is detected outside of the 3 *μ*m radius, it counts as a false positive, while false negatives are neurons that have not been detected. For a 150 x 400 pixels image of 100 neurons with 50 true positives, the number of true negatives would be all the remaining 59,900 pixels, making TP a skewed class. Given this resulting unbalanced data set, true negatives are not taken into account in our tables nor in the accuracy formula. Table 1 reports the number of TP, FN, and FP, their respective rate, and the resulting accuracy for automatic detection. The results with data sets 1 and 2, both acquired with a spinning disk, are in line with other detection algorithms, allowing the automatic detection of most of the neurons. The result with data set 3 is affected by the acquisition technique (light-sheet microscopy), and the lower signal-to-noise ratio compared to the first two. However, in such recordings, missed spots may be manually added through a dedicated interface (see Tracking section). The annotation may be done on one frame, and See Elegans will use the neighboring tracks to reconstruct the trajectory of the missed neurons. Data set 2 was also processed with two other publicly available tracking pipelines to compare the accuracy of the algorithms in detecting neurons in a still volume. Again, we first determine ground truth through manual annotation and then count the number of TP, FP, and FN as previously described.

**Table 1:** See Elegans’ automatic detection performance for 3 data sets. The table shows the number of correctly (TP), undetected (FN), and incorrectly detected (FP) neurons, their rates, and accuracy for each data set. See Elegans’ performance is higher for data sets 1 and 2 (obtained with 60x confocal microscopy) compared to data set 3 (acquired with light-sheet microscopy). These results are consistent with other detection algorithms and enable automated detection of most neuronal spots, with the ability to manually add missed regions.

	TP	FN	FP	TP r.	FN r.	FP r.	Acc.
**data 1**	91	44	18	0.67	0.33	0.13	0.59
**data 2**	93	29	10	0.76	0.24	0.08	0.70
**data 3**	44	54	17	0.45	0.55	0.17	0.38

**Fig 3:**
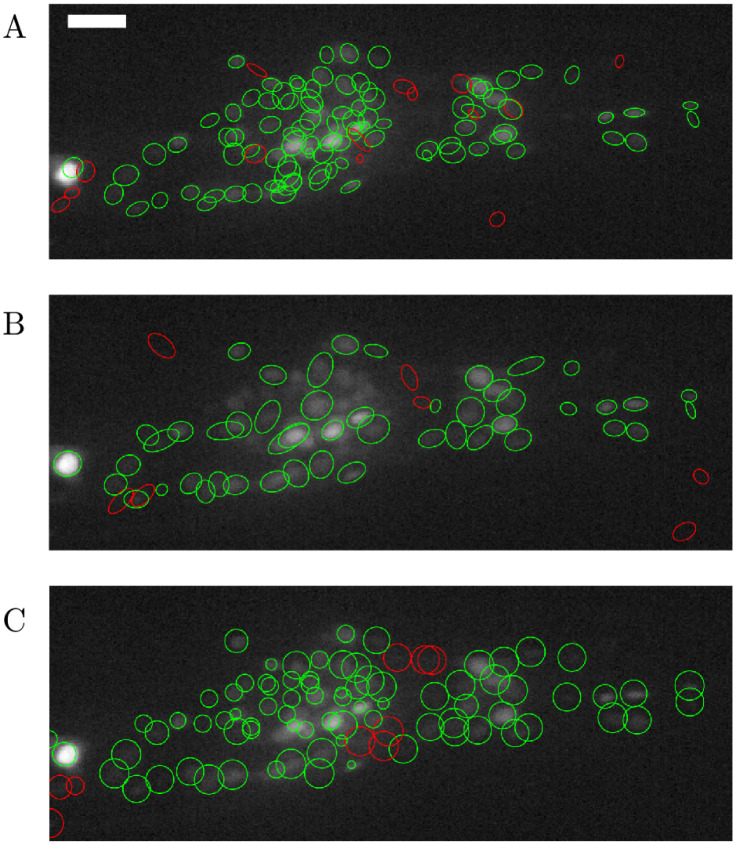
Comparison of detection results. The figure shows for the same acquisition the results of automatic segmentation in the case of See Elegans (panel A) and two other tracking algorithms: RoiEdit3D (panel B), Trackmate (panel C). Green circles represent true positives, while red circles represent false positives. The white bar corresponds to 10 *μ*m.

**Fig 4:**
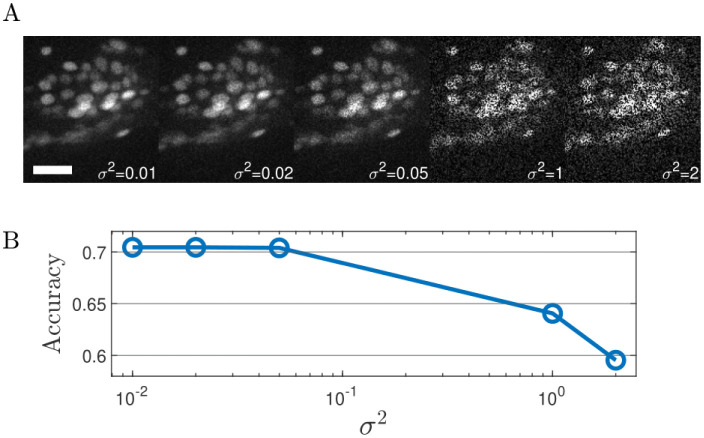
ROI with increasing variance of multiplicative noise and resulting accuracy. Panel A shows different images of the same ROI from data set 2 with an increased variance of multiplicative noise (*σ*^2^) from left to right. The length of the white bar represents 10 *μ*m. Panel B shows the resulting plot of the accuracy as a function of noise variance: it is initially relatively stable and then decreases for higher values of noise from about 0.70 to about 0.60.

Fig 3 presents a comparative analysis of tracking algorithms (See Elegans, TrackMate [30], and ROIedit3D [14] respectively) applied to a static slice from data set 2. The radius and threshold parameters used for blob detection in TrackMate were selected from a range between 4.75–5.75 and 7.5–8.5 respectively, to maximize accuracy. For ROIedit3D we chose the confocal parameter set and filtered the results based on the area of the spots (> 300 pixels) and the ratio between the major and minor axes (> 3). Green circles represent true positives, while red circles represent false positives. Table 2 reports the result of the comparison, the number of TP, FN, FP, their rates, and accuracy. The comparison indicates that See Elegans outperforms TrackMate, based on the same method as the first one (convolution through laplacian of gaussian), and ROIedit3D, whose detection process is based on image thresholding and strongly relies on intense signals, as the ones associated with the RFP channel for example. See Elegans has a higher amount of detected neurons and a lower amount of false negatives and false positives. We also tested the performance of our algorithm in the presence of multiplicative noise added to the image. We reproduced increasing amounts of noise levels as increasing variance (*σ*^2^) of multiplicative noise and ran the automatic detection step of the algorithm on the noisy images from data set 2. We then calculated the percentage of true positives, false negatives, and false positives. Table 3 reports the number of TP, FN, and FP for different noise levels. Fig 4 shows an ROI from data set 2 with increasing multiplicative noise variance and the resulting accuracy. As the data show, the performance is initially relatively stable and then decreases for higher values of noise, with an increase in the number of false negatives, and a decrease in the number of true positives. We also calculated the computational time required by the algorithm to perform tracking. Table 4 shows the computational time per volume, calculated for different volume sizes. With these values, it takes about 7 minutes to segment a 10-minute long recording acquired at 3 volumes per second, with a 165 x 440 x 12 voxels volume size.

**Table 2:** Comparison of automatic detection performance for different algorithms. The table shows the number of correctly detected neurons (TP), undetected ones (FN), and false detections (FP), their rates, and the accuracy of the output of See Elegans and two other tracking algorithms: Trackmate and RoiEdit3D. The parameters of See Elegans and TrackMate include threshold and spot size settings, while ROIedit3D was set to the confocal parameter set. See Elegans detects more neurons and has fewer false negatives and positives, outperforming other algorithms.

	See Elegans	TrackMate	RoiEdit3D
**TP**	93	80	49
**FN**	29	42	73
**FP**	10	9	7
**TP r.**	0.76	0.66	0.40
**FN r.**	0.24	0.34	0.60
**FP r.**	0.08	0.07	0.05
**Acc.**	0.70	0.61	0.38

**Table 3:** Performance of the algorithm in the presence of noise. Percentage of true positives, false negatives, and false positives with increasing variance of multiplicative noise (*σ*^2^) applied to a volume before the automatic processing through See Elegans. The increasing level of noise increases the number of false negatives and decreases the number of true positives, thus affecting the accuracy.

*σ* ^2^	TP	FN	FP	TP r.	FN r.	FP r.	Accuracy
0.01	93	29	10	0.76	0.24	0.08	0.70
0.02	93	29	10	0.76	0.24	0.08	0.70
0.05	88	34	3	0.72	0.28	0.03	0.70
1.00	82	40	6	0.67	0.33	0.05	0.64
2.00	78	44	9	0.64	0.36	0.07	0.59

**Table 4:** Computational time with respect to volume size. The table shows the computational time required to spot neurons for increasing sizes of volumes. For example, for a 165 x 440 x 12 voxel volume recorded for 10 minutes at 3 volumes per second, the run-time of the detection process is about 7 minutes.

Volume Size	Comp. time (s)
**165 x 440 x 12**	0.24
**130 x 350 x 10**	0.19
**100 x 260 x 7**	0.16
**80 x 220 x 6**	0.14
**66 x 176 x 5**	0.13

Overall, our comparison shows that the accuracy of our algorithm in the detection process is higher than that of already available algorithms and that it is also relatively stable with respect to noise, while the processing run-time is at a reasonable level. Finally, although blob detection may be put in a parallel form, See Elegans does not have high requirements in terms of computational power and may run on a desktop PC.

It may be argued that a properly trained Mask-RCNN model might provide higher accuracy in neuron segmentation. While we acknowledge the power of deep learning approaches, we believe there are several aspects that make See Elegans a valuable tool.

Requiring less computational resources compared to deep learning models, See Elegans is more accessible to users working with large data sets and those with limited hardware resources. Its use of rule-based techniques provides greater interpretability than deep learning models and avoids the dependency on a training data set, making it useful in scenarios where data annotation is costly or impractical. It also provides considerable flexibility, allowing users to configure parameters to their specific needs. Finally, as demonstrated in Table 1, See Elegans delivers competitive accuracy on our data sets. While a well-trained Mask-RCNN model might theoretically offer higher accuracy, achieving such results requires significant computational resources and may not necessarily ensure superior performance in all scenarios.

### Tracking

**Fig 5:**
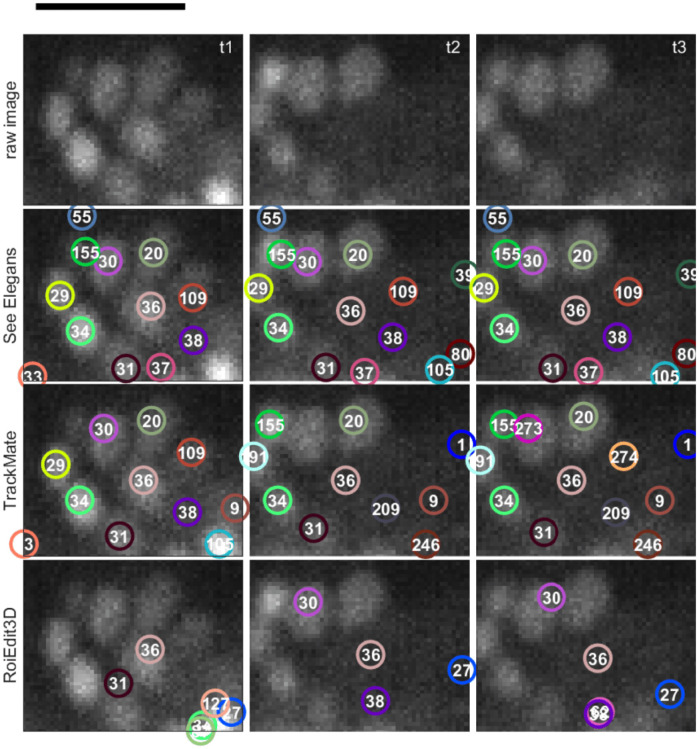
Tracking of ROIs falling under detection threshold displayed in temporal order. The top row shows raw data, while the following rows report the tracking results for See Elegans, TrackMate, and ROIedit3D. At t1, neuron 30 is visible in the ROI center, and See Elegans tracks it up to t3, while TrackMate loses it at t2 and ROIEdit3D at t1. The black bar represents 10 *μ*m.

**Fig 6:**
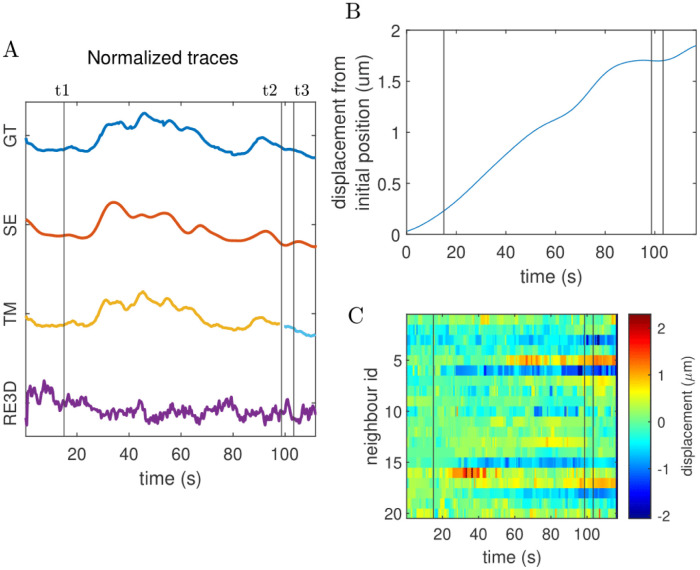
Resulting traces of neuron 30, falling below the detection threshold. Panel A shows the traces for ground truth (GT), See Elegans (SE), TrackMate (TM), and ROIedit3D (RE3D). See Elegans captures the dynamics of GT, but TrackMate and RoiEdit3D present gaps and/or artifacts. TrackMate assigns two different IDs to the same neuron, hence the different colors. Panel B reports the absolute displacement of neuron 30 from t1 onwards. Panel C reports the difference between the distance of neuron 30 from 20 of its closest neighbors at t1 and at subsequent times. The plot reveals some neurons moving closer and some moving away with an excursion up to 2 *μ*m (e.g., rows 6 and 16).

Fig 5 highlights the advantages of the tracking step in our proposed algorithm by presenting a representative crop from data set 1. This crop features a neuron that falls below the detection threshold, a scenario that often poses challenges to tracking algorithms. The figure compares the results obtained from See Elegans with those from two other algorithms, TrackMate and ROIedit3D. This particular neuron was selected to exemplify the efficacy of See Elegans in restoring tracks of neurons that slip beneath the detection threshold. The reported ROIs are from time t1, t2, and t3, corresponding to seconds 15.0, 33.0, and 73.33 from the start of the recording respectively. At times t1 and t3, neuron 30 has a relatively good level of activation (above threshold), while at time t2 it shows a lower signal. Because of this, it is hardly detectable at t2 and is therefore difficult to track throughout the indicated times. As the figure shows, while See Elegans is able to track the neuron, TrackMate separates its trajectory into two segments, losing the information in the intervening time gap and assigning two different IDs to the same neuron. Instead, ROIedit3D is able to follow the neuron lost by TrackMate but is affected by the noise, and thus its performance seems to be compromised, as clearly evident from the corresponding trajectories reported in Fig 6. As a result, the fluorescent trace obtained by manually linking the incomplete segments associated with the same neuron in Trackmate (using different IDs), still has a gap, while the trace obtained with ROIedit3D is uninterrupted, but also affected by artifacts. It is worth noting that the specific neuronal track of neuron 30 may be retrieved in TrackMate with a different set of parameters, for example by lowering the detection threshold, extending the time gap for spot linking, or changing the size of the filter. However, these changes would affect the overall detection and tracking performance, not necessarily in a better way. In fact, a lower threshold would result in more false positives, while a longer time gap for segment linking may result in a higher number of wrong linkages between tracks of different neurons. In the given case, the parameters used in TrackMate were manually optimized to obtain the best results for the whole recording. In addition to the resulting fluorescent traces (panel A) for the recording shown in Figs 5 and 6 reports the absolute displacement of the central neurons (panel B), together with the absolute distance variation in time for 20 of its closest neurons (panel C). In particular, panel B shows that neuron 30 covers a distance comparable to its own size in less than two minutes. Being neuron 30 located in a densely packed area, such a movement is sufficient to produce artifacts. However, the mutual distance inference method allows the code to keep track of this neuron despite its significant displacement. Panel C reports the time variation of the distance between neuron 30 and the twenty closest neighbors used to infer its position. As the color map shows, some neighbors move closer while others move away with respect to the untracked neuron (displacements up to 2 *μ*m in 100 seconds). The presence of such elastic deformations in the neuronal arrangement may further hinder the tracking process, making the use of position inference techniques, such as the one implemented in See Elegans, crucial.

### Identification

**Fig 7:**
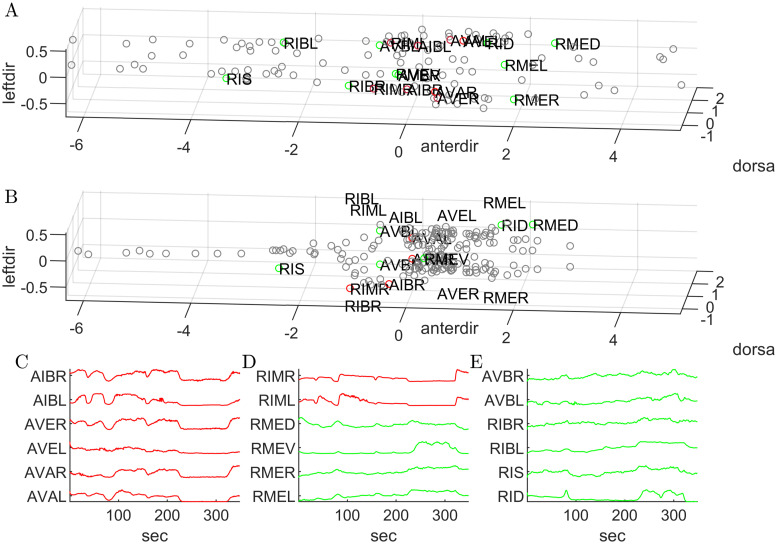
Result of the identification process on data set 2. Panel A reports the detected neuronal positions rotated to align with a coordinate system in which the x, y, and z axes correspond to the anterior, dorsal and left directions respectively. Panel B shows the spatial arrangement of neurons according to the model. The axes are normalized to the average distance of the 6 initially identified neurons, and the origin is located at the mean point of these neurons. Panels C, D, and E report the trace of the two anti-correlated sets of the neurons, promoting backward (red curves) and forward movement (green curves) respectively.

We also assessed the performance in the identification process, as reported in Fig 7. This figure shows the results of the neuronal classification when applied to data set 2. Following the segmentation and tracking procedures, we cleaned the traces relative to the acquisition, which were then used to automatically determine the individual cell identities. The software’s identification process is underpinned by two distinct neuronal groups that respectively promote backward and forward movements. These groups were successfully segmented and tracked in the preceding steps for data set 1. In particular, we checked the presence of the AVA neuronal class, as it is crucial for the identification because it displays one of the highest correlations among all neurons and a stereotyped position in the nematode’s head. However, it is unlikely to miss these neurons as their fluorescent intensities are usually among the highest ones.

Comparing Panel A with Panel B, representing the model distribution based on [5] and the result of the identification step respectively, the neuronal position seems to be consistent, despite showing a general variability in the neuronal arrangement. Also the fluorescent traces (panel C, D, and E in Fig 7) associated with the resulting neuronal class identities are consistent with the expected dynamics of the forward- (RME, RIM, AVB, RIB, RIS, and RID classes, whose traces are colored in green) and the backward-promoting neuronal groups (AIB, AVE, AVA, and RIM classes, colored in red), meaning that they show relatively high intra-group correlation and high inter-group anticorrelation. To better show this aspect, we ran See Elegans on data set 4 and processed the neuronal traces according to [28] to show that the resulting analysis is in line with previous findings regarding the global dynamics of the nervous system and the specific activity of the AVA neuron. This analysis is included in the S1 File.

To address situations where neurons exhibiting similar activity patterns are closely located, as in the case of AVEL/R and AVAL/R, the algorithm refers to the reported connectome with particular attention to maintaining the anterodorsal and left/right order of the cells. A geometric control is also performed: the choice of left/right neurons is derived from the position of the head, indicated by the user, and the location of backward-promoting neurons, which have high and correlated activity, thus easily visible. Thanks to these reference points, the anteroposterior, dorsoventral, and lateral directions are identified (see Fig 4 in S1 File). These directions are then aligned with the reported connectome [5], and neuron identities are assigned, treating the problem as a linear assignment problem. To quantify the performance of the algorithm in assigning neuronal identities, we applied See Elegans to the 5 data sets in which identification has also been manually performed following the procedure detailed in [28, 34, 35] (See Table 5). The data sets comprise data set 1, data set 2, and 3 additional data sets (data sets 4, 5, and 6) of unstimulated nematodes, all of which underwent minimal manual correction to include all identifiable neurons. From the comparison with the manually annotated data, the true positive rate of the automatically identified neurons is 65%, reaching 90% in the case of backward-promoting neurons. The forward-promoting neurons are characterized by smaller somas and thus weaker signals which make them more difficult to identify and segment. This is particularly evident in data set 6, where such neurons have a lower identification rate with respect to the other ones. However, in all of them, the algorithm not only successfully identified the dorsal and lateral directions, but it also provided at least 3 backward-promoting neurons for both the left and right side of the animal. This is particularly helpful considering that, with an interface that highlights positive and negative activity correlations of detected spots and the anteroposterior, dorsoventral, and lateral axes while inspecting the neuronal recordings, the user can easily identify backward and forward-promoting neurons respectively.

**Table 5:** True positive rates of the automatic identification. The table shows the true positive rates reached by the algorithm in assigning the neuronal identities to data sets 1,2, and 4–6. The low values of data sets 6 are associated with low signals of the targeted neurons and/or deformation in their typical arrangement.

Data	TP
**Data set 1**	0.80
**Data set 2**	0.64
**Data set 4**	0.80
**Data set 5**	0.62
**Data set 6**	0.40

## Discussion

We introduce See Elegans, an algorithm for processing volumetric calcium imaging recordings of paralyzed *C. elegans* strains with pan-neuronal calcium indicator expression, which we successfully applied to process multiple acquisitions. Its intuitive interface enables tracking result inspection in 2D and 3D, parameter adjustment, and manual correction of segmentation, tracking, and identification results.

The detection step utilizes common methods, such as 3D laplacian of Gaussian filtering of volumes, with user-adjustable parameters. The tracking step combines techniques such as the Runge-Kutta solution for spot linking, segment linking based on average distance preservation, and distance preservation between spots for inferring neuron positions [28, 30]. For the tracking step, other algorithms rely on the RFP channel which typically exhibits constant fluorescent activity, whose time variations are mainly due to two phenomena: bleaching or movements. Besides simplifying segmentation and tracking, the information in the RFP channel is especially suited to eliminate artifacts caused by the latter phenomenon, but at the cost of requiring more complicated acquisition setups and a strain with RFP and GCaMP expression. Instead, our solution may be used to process recordings of paralyzed nematodes acquired in the GFP channel only.

Furthermore, while our demonstrations primarily utilized microfluidics imaging chips, the algorithm is designed to be equally effective with other standard practices, such as mounting *C. elegans* on a thin layer of agarose between glass coverslips, as long as the worm remains sufficiently immobilized and the optical requirements are met. As the results on data sets 1, 2, and 3, and the noise level analysis show, the optical requirements for robust results include a magnification of at least 40x and a numerical aperture above 1, as well as an acquisition rate that is able to capture the subtle movements and deformations in the *C. elegans* arrangement (> 3 volumes per second).

It is also important to notice that while we only tested two versions of the same calcium indicator (GCaMP6s and GCaMP6f), other calcium indicators are expected to be compatible with See Elegans, as long as they provide clear representations of neuronal dynamics without significantly affecting the signal-to-noise ratio (crucial for segmentation), as the identification algorithm focuses on the coherence of signals rather than their specific shapes. However, given the dense arrangement of the neurons in the head of *C. elegans*, the fluorophores need to be localized in the nucleus to avoid overlapping signals and reduced precision.

A novel feature of See Elegans is its automatic identification of a subset of neurons with stereotyped activity, based on signal correlation properties and spatial arrangements, a procedure that has been previously applied manually [28, 34, 35, 37].

The algorithm reaches an overall true positive rate of 65%, which goes up to 90% in the case of backward locomotion-promoting neurons (for which the algorithm always succeeds in finding at least six representatives) which provide the main contribution to the brain dynamics in unstimulated animals, and offers an interface to inspect and manually adjust the results, while highlighting correlations with such neurons to facilitate the identification of correlated and anti-correlated neurons, as the ones of the forward-promoting set. Future releases of the algorithm may leverage the stereotyped activity of other neuronal groups, such as those activated during animal turning, or motor neurons.

It is worth noticing that, while the algorithm significantly automates the detection, tracking, and identification of neurons, user input is required for initial parameter setting in segmentation, tracking, and identification steps. This input is crucial for adapting the algorithm to specific data sets and experimental conditions (e.g.: a different magnification). Once these parameters are set, the algorithm operates automatically, processing the volumetric calcium imaging recordings without further human intervention. Finally, after the automatic steps, users have the possibility to inspect and, if necessary, manually adjust the segmentation, tracking, and identification outcomes at any point.

In conclusion, See Elegans integrates various techniques for cell segmentation, tracking, and identity assignment. Its user-friendly interface allows parameter customization and result inspection, accelerating the whole procedure while ensuring accuracy.

## Materials and methods

### Calcium imaging setup

To record calcium imaging signals for data set 1 we used an inverted microscope (IX73, Olympus, Tokyo, Japan) connected to a spinning disk (Xlight V-3, CrestOptics, Rome, Italy), a 60x water immersion objective with a 1.2 numerical aperture (UPLSAPO60XW, Olympus, Tokyo, Japan), and an EMCCD camera (Evolve 500, Teledyne Photometrics, Tucson, Arizona) with exposure time at 10 ms. The excitation light (470 nm) was provided by a laser (LDI, 89 North, Williston, Vermont), while a piezo-stage (Mad City Labs, Madison, Wisconsin) was used to focus on different planes. Image acquisition was managed by MetaMorph software and the camera shutter was synchronized through TTLs with the motion of the piezo stage, which followed a sawtooth trajectory with a plane spacing of about 2 *μ*m. Given the exposure time and the time needed to reach the next acquisition height (5 ms), a new plane is acquired every 15 ms.

For data set 2, the optical setup involved a Spinning Disk Confocal with a 40x water objective and 2x2 binning. The excitation was achieved using a 488 nm laser, with an exposure time set to 30 ms. The imaging rate was established at 3 volumes per second.

For data set 3, the nematode was imaged using a Dual Inverted Selective Plane Illumination microscope (Applied Scientific Instrumentation, USA). A water-immersed 0.8 NA 40× objective (Nikon, USA) was used for 5 minutes, with illumination interleaved between a 488-nm laser at 5 mW power and a 561nm laser at 5-mW power. Volumetric stacks (41 slices, voxel size 0.1625 μm × 0.1625 μm × 1 μm) in two colors were obtained at two volumes/second for 5 minutes, producing 49,200 images per session.

### Microfluidic device

To perform calcium imaging for data set 1, nematodes are confined in microfluidic devices. Through the soft-lithography process [50], we produce a modified version of the olfactory chip described in [51], with a slightly rotated loading channel compared to the original one. All other channels (for inflow, outflow, flow control, and stability) are the same as in the original. The substrate of the chip is bonded to a 170-micron thick microscopy cover slip to allow optical access to high-magnification objectives. Tygon tubes containing nematodes are directly connected to the chip, while the tubes needed for solution injection and outflow are connected through cannulas.

### *C. elegans* culture and strains

The *C. elegans* strain used for data set 1 imaging tests is the Mzmls52 ([mzmEx711 = [unc-31::NLSGCaMP6f cod opt intron] complex array 2.5ng/ul] 9x OC on X 16x OC on I-V), which was a kind gift from Dr. Manuel Zimmer (University of Vienna, Vienna, Austria). This strain was cultured at 20°C on nematode growth medium (NGM) plates seeded with *Escherichia coli* OP50 as a food source (Brenner, 1974). For data set 2, the employed strain was FC036 (pyIs(Punc-31::NLS::GCaMP6F); lite-1), which exhibits nuclearly localized expression. For data set 3, imaging experiments were conducted using the transgenic strain QW1217 (zfIs124[Prgef-1::GCaMP6s]; otIs355[Prab-3::NLS::tagRFP]), which expresses pan-neuronal somatic GCaMP6s and nuclear-localized Red Fluorescent Protein (RFP) in all neurons. *C. elegans* were cultivated at 20°C on NGM with *E. coli* OP50 as a food source.

### Sample preparation

Prior to experiments, young adult *C. elegans* are picked from a synchronized culture and put in a 35 mm Petri dish with *E. Coli*. 5 minutes before the start of the experiments, one young adult animal is put on a 35 mm NGM Petri dish (without feeding bacteria) for a minute and then immersed in a drop of levamisole (diluted at a millimolar concentration in sBasal). After 1–2 minutes the worm is loaded onto the olfactory chip through a syringe and a Tygon tube, and placed on the piezo stage for acquisition. In preparation for imaging data set 2, Day 1 adult hermaphrodite *C. elegans* were starved for 20 minutes to 1 hour. The animals were previously conditioned in a long-term memory assay, placed in 5 mM tetramisole for 10 minutes, and then put on an imaging chip with an s-curve. Each animal was stimulated with 111 *μ*M butanone (BTN) in this pattern: 1 minute OFF, 30 seconds of 1-second pulses of BTN, 30 seconds OFF, then repetition of a 30 seconds ON/OFF cycle 4 more times, then 1 minute OFF at the end. For data set 3, young adult hermaphrodite *C. elegans* were encapsulated within a permeable hydrogel. After immobilization through immersion in 5 mM tetramisole for 1—2 minutes, worms were placed into a 3 μl of a solution of 13.3% polyethylene glycol diacrylate (Advanced BioMatrix, USA) with 0.1% Irgacure (Sigma-Aldrich, USA) on a glass coverslip between two spacers 200 μm in height. Through ultraviolet light, the hydrogel was hardened, encapuslating the worm, and secured to a glass slide with low viscosity (cyanoacrylate ester, Permabond, USA). This setup was mounted in a 50-mm Petri dish with vacuum grease filled with 50 ml of S-Basal solution and 5 mM tetramisole for imaging.

### Data sets

We applied the proposed algorithm to three data sets. Data set 1 was acquired at the Center for Life Nano- and Neuro-science of the Italian Institute of Technology in Rome with the optical setup described in this section and is a 17-minute long recording of a paralyzed worm (Mzmls52 strain).

The second data set was acquired at the University of California in San Francisco. It is a 10-minute-long recording of a paralyzed worm.

Data set 3 was acquired at the Physiology & Biophysics department at Boston University and belongs to a 5-minute light-sheet imaging of the head of a *C. elegans*.

#### Pre-processing

We applied an image Kalman filter to pre-process the data. For each data set, we subtracted a frame-specific background by taking a dark square crop of the image.

### Algorithm implementation

All computational processes are performed through MATLAB software (Natick, Massachusetts, USA) on a desktop (3.60GHz Intel Core i5–8600K with 16 GB of RAM, Windows 10 64bit). Data set 1 and 2 were preprocessed through an image Kalman-Filter to remove noise, while data set 3 with a median filter. The results of the processing may be visualized through Matlab or through ImageJ.

#### Blob detection

Nuclear regions are identified by applying a Laplacian of Gaussian filter in 3d and then by thresholding the element-wise product image of the local maxima and the original image. The user is asked to set the size of the filter, the sigma of the Gaussian used in the filter, and the intensity threshold under which blobs are discarded. These parameters may be adjusted to match the magnification used in the experiments and in particular the size of the neurons. For further details, see the documentation of the GitHub repository.

#### Track segment creation

Detected blobs on different frames are linked from frame to frame by treating the problem as a linear assignment problem (LAP), as done in [30, 31]. To solve the problem, we use the MATLAB *matchpairs* command, which applies the procedure described in [52]. The user is asked to set the maximum distance for frame-to-frame linking (in micrometers). Any connection higher than this distance is prohibited. As in [30], the cost for interframe linking between two blobs is given by the square of the distance between them, and the alternative cost is set as 1.05*cmax, where *cmax* is the maximum linking cost. The user is also asked to set a maximum time gap over which spots may not be connected.

#### Whole track creation

To build tracks, segments created through frame-to-frame linking are further linked by treating the task as a linear assignment problem (LAP) again, as in [30, 31]. The user is asked to set the maximum time delay to link two segments (in seconds) and the maximum distance (in micrometers). Links exceeding any of these two values are not allowed. As in the previous step, we use the *matchpairs* MATLAB function, and the cost for each possible link is calculated as the square of the distance between a segment end point in time and a segment start point in time. The alternative cost is calculated as before, equal to 1.05*cmax, where *cmax* is the maximum linking cost.

#### Track restoration

As an additional step with respect to [30, 31], we retrieve some links of unconnected segments exploiting the consistency of the distance between neurons in different frames. In this step, we keep track of the average distance of a single segment with respect to the other ones and use this information to find the best candidate for segment linking. To do this, we simply calculate the mean displacement of an unconnected track with respect to all others in all available frames and then look for a tracked segment that has the same distance ratios in other frames. The segment that has the lowest difference in its mean displacement with respect to the other tracks is then linked to the initial segment. As in the previous steps, there is a maximum distance for segment linking and a maximum time gap that are user-defined. A higher parameter will track neurons that undergo higher deformations in the recording, extending the search radius of the neuron for cross-frame linking.

#### ROI segmentation

Once the tracks are defined, each ROI at each time is segmented. As an initial step, we take a 3D ROI that extends for about 6 um around the blob. We then clean the image by filtering it with a gaussian filter of different sizes and applying a threshold to obtain a black-and-white image. We then take the result that best divides the blob from its surrounding ones keeping a size of the blob of about 2 *μ*m. After this, we apply an ellipsoid fit of the 3D segmented volume. This allows reaching a sub-pixel localization of the spots and an approximate outline of the cellular nuclei, and it also identifies major axes of extent of the blob together with their orientation. Once obtained, axes and orientation are fixed throughout the trajectory at the average of the results obtained for different time points weighted by the intensity of the ROI at that time point.

#### Trace extraction

Once the blobs are segmented and linked in tracks, we extract the signal of the neurons by taking the 90% brightest pixels inside the neuronal ROI of the background-subtracted image and subtracting to it a 1-pixel-thick corona around the ROI. The obtained signals are then smoothed through an average filter, whose parameters may be set by the user.

#### Identification

The identification process starts by looking for two pairs of three roughly collinear points arranged along the spatial direction with the largest spatial distribution of neuronal positions (found through PCA). The research of these points considers only the combination of neurons with the highest correlations among all of the members of a set of 6 with two subgroups of up to 3 neurons that are roughly collinear and parallel to the first principal component. After finding these neurons, the mean point in 3D and the mean distance among all of the neurons belonging to the identified subset are calculated. At this point, the user needs to specify the direction of the head, which will be used to distinguish left and right directions. The dorsal direction is estimated by summing the number of neuronal spots (from the mean point of the six neurons initially identified). The side of the plane passing the closest to all six neurons containing the highest number of neurons is identified as the dorsal side. Once the dorsal, anterior and left directions have been assigned, all of the neuronal coordinates are rotated to a new coordinate system based on these directions. The coordinates are then divided by the average distance among the six identified neurons to compensate for scale differences and the origin of the coordinate system is set to their mean point. The neuronal arrangement obtained is then compared with a model distribution based on [5] and on observations made during pan-neuronal calcium experiments. Neuronal spots of the model are assigned to the observed ones by treating the task as a linear assignment problem, solved through the *matchpairs* algorithm of MATLAB. The cost function between two neurons takes into account not only their position but also their correlation. If *C* is the correlation between two neurons and *C*_*μ*_ and *C*_*σ*_ are the mean and standard deviation of all correlations respectively, then three additional groups are specifically looked for: neurons that show high correlation (*C* > *C*_*μ*_+ *C*_*σ*_), and high anti-correlation (*C* < −*C*_*μ*_ − *C*_*σ*_ < 0) with the first identified neurons.

## Supporting information

S1 File (DOCX)
