# Supplementary material for: See Elegans: Simple-to-use, accurate, and automatic 3D detection of neural activity from densely packed neurons
Source: PLoS One. 2024 Mar 22;19(3):e0300628. doi: 10.1371/journal.pone.0300628 (PMC10959381; doi:10.1371/journal.pone.0300628)
Supplement: S1 File — (DOCX) [file pone.0300628.s001.docx]

**Supplementary Material**

Global brain analysis and AVA dynamics representation through PCA.

To show how the characteristics of neuronal activity are maintained through See Elegans’ cell segmentation and tracking steps, we processed the activity traces of data set 4 according to the procedure described in [1], which demonstrates that the neuronal signals can be reduced in dimensionality through PCA decomposition. In particular, the first principal components (1-3) are guided by the activity of the backward-movement promoting neurons (with AVA as the main contributor) and their anti-correlated neuronal set (promoting forward locomotion).

To illustrate how neuronal activity traits are preserved during C. elegans’ cell segmentation and tracking, we applied the method outlined in [1] to dataset 4's activity traces. This involved using PCA decomposition to reduce the dimensionality of the data. As found in [1], the first principal component is primarily influenced by backward-movement neurons (with a high contribution confirmed to be coming from the AVA, AIB, AVE, and RIM neurons) and their counterparts that promote forward locomotion (see Figure 1), with negative weights. Figure 2 - panel A displays the time-evolving trajectory of the first three components. As shown by the figure, the trajectory results to be cyclical, as in [1], reflecting the temporal evolution of neuronal states. Furthermore, we highlighted the role of AVAR neurons during various trajectory phases (rise, high, fall, low) obtaining the same distinct phase space separation. For clarity, Figure 3 presents AVAR’s fluorescence trace and its activity state classification.

These data confirms that the overall results of the segmentation, tracking and identification of See Elegans produce observations in line with previous findings for global nervous dynamics and for the example case of AVA activity.

[1] Kato, Saul, et al. "Global brain dynamics embed the motor command sequence of Caenorhabditis elegans." Cell 163.3 (2015): 656-669.

**Figure 1, Loadings of PC1 and role of locomotion promoting neurons**. The bar plot shows how much every neuron contributes to PC1. Red bars refer to backward movement-promoting neurons, while green ones refer to the forward group. As found in [1], the PC1 has the main positive loadings coming from the AVA, AVE, AIB and RIM neurons, while the negative weights are associated to AVB and RME neurons.


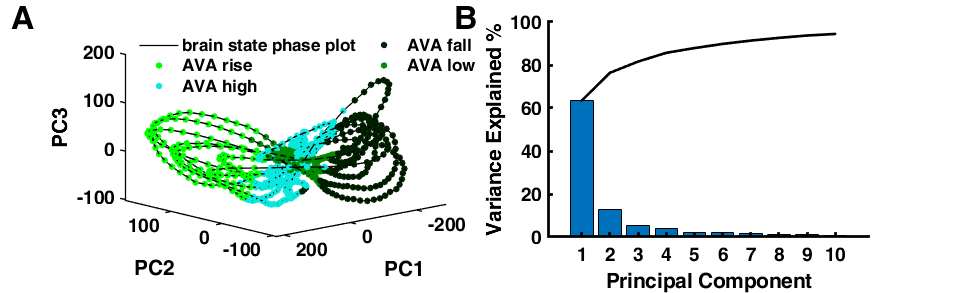


**Figure 2, Global Brain Analysis and AVA phases highlighted**. Panel A reports the trajectory of the neuronal state represented through the first three components of the PCA, as in [1]. Additionally, the rise, high, fall and low states have been highlighted on the trajectory. Panel B reports the percentage of the variance explained by the components of the PCA, showing that the first three components explain more than the 80% of the variance of the data.


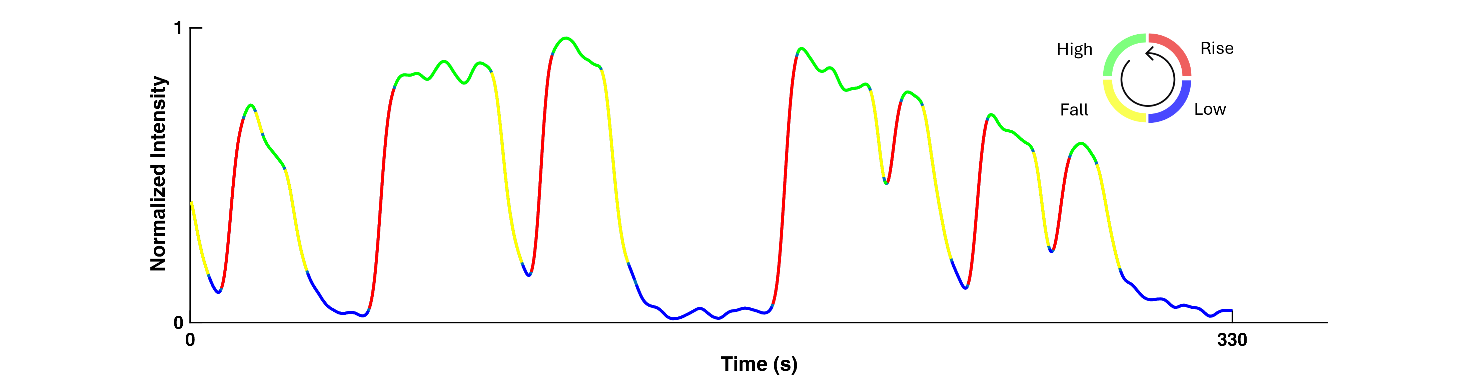


**Figure 3, AVA phases**. The plot reports the fluorescence trace of the AVAR neuron with the highlighted rise, high, fall, and low phases according to the legend.

Identification process:

The identification process starts with the localization of two symmetrical pairs of 3 collinear neurons with high signal and high correlation, which are to be identified as belonging to the set of backward locomotion promoting neurons. The success rate of this process in our tested datasets was 100%. Once these are identified, the dorsoventral direction is derived from their location, while the anteroposterior direction is assumed to be as the PC1 of the coordinates representing neuronal locations. Finally, the lateral axis is derived from the first two. The following figure represents the result of this first step of identification. Green dots represent the dorsal neurons, while the blue ones represent the ventral neurons. Additionally, a plane highlights the region with the most probable presence of the most active backward promoting neurons (AVA, AVE, and AIB/RIM). Inferring the anteroposterior, dorsoventral, and lateral directions is crucial to distinguish between closely located and highly correlated neurons.


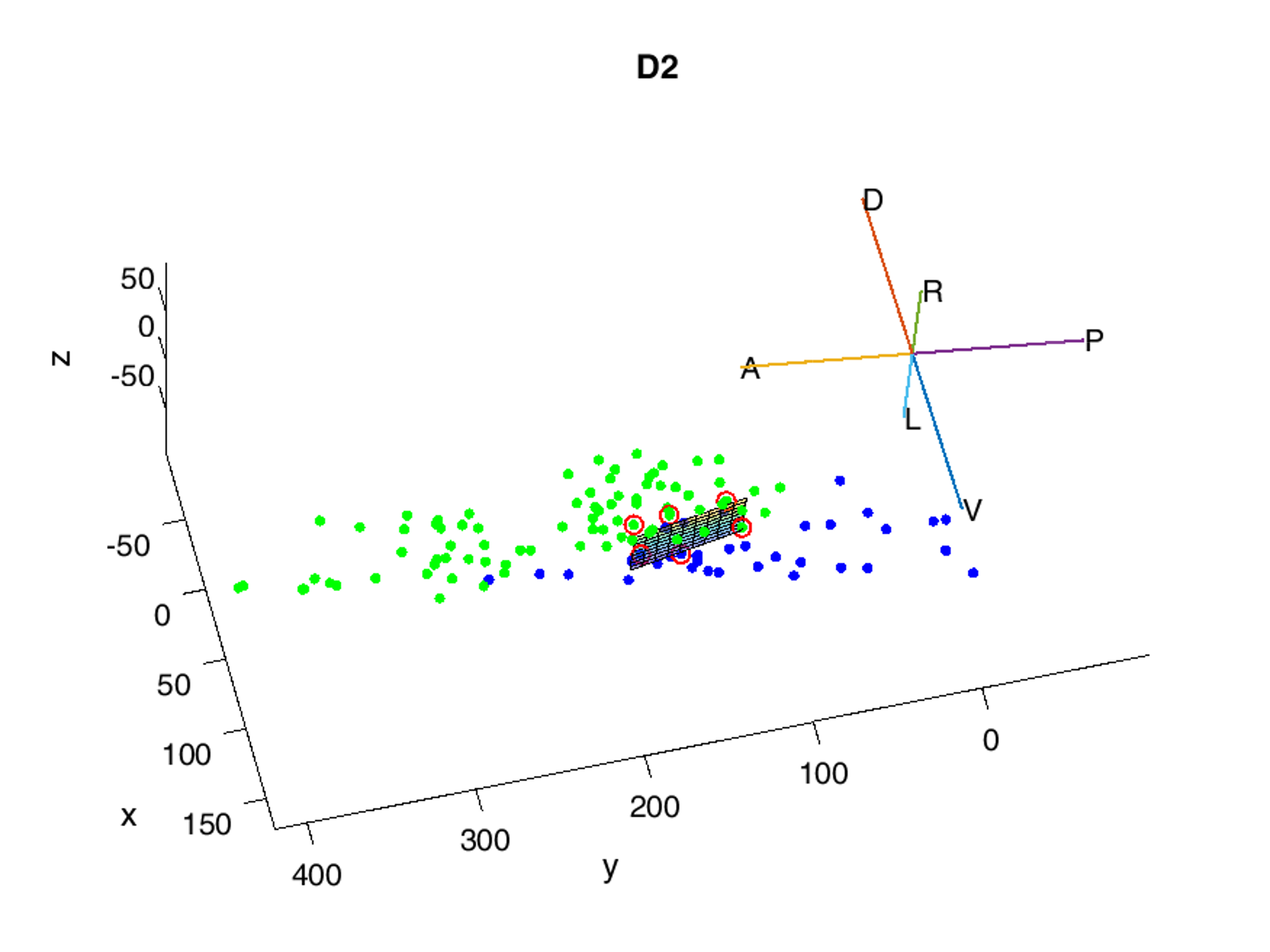


**Figure 4, Identification of anteroposterior, dorsoventral and lateral axes from the activity and the arrangement of neurons**. The figure represents the result of the first step of the identification process on data set 2, with the body directions marked as (A: anterior, P: posterior, L: left, R: right, D: dorsal, V: ventral). The anteroposterior axis is derived as the PC1, while the dorsoventral is derived from the position of the backward-promoting neurons (such as AVA). Once these are identified, the lateral axis is derived from the first two. The green dots in the image represent the dorsal neuorns, while the blue dots represent the ventral ones. Additionally, a plane highlights the region with the most probable presence of the most intense backward promoting neurons (AVA, AVE, and AIB/RIM).
